# Isolation and characterization of two homolog phages infecting *Pseudomonas aeruginosa*

**DOI:** 10.3389/fmicb.2022.946251

**Published:** 2022-07-14

**Authors:** Niu Yuanyuan, Yang Xiaobo, Wang Shang, Yang Yutong, Zhou Hongrui, Li Chenyu, Xue Bin, Zhang Xi, Zhao Chen, Shen Zhiqiang, Wang Jingfeng, Ling Yun, Yu Pingfeng, Qiu Zhigang

**Affiliations:** ^1^College of Marine Ecology and Environment, Shanghai Ocean University, Shanghai, China; ^2^Key Laboratory of Risk Assessment and Control for Environment and Food Safety, TianJin Institute of Environmental and Operational Medicine, Tianjin, China; ^3^College of Environment and Resource Sciences, Zhejiang University, Hangzhou, China

**Keywords:** bacteriophages, *Pseudomonas aeruginosa*, genome, tail fiber protein, phage therapy

## Abstract

Bacteriophages (phages) are capable of infecting specific bacteria, and therefore can be used as a biological control agent to control bacteria-induced animal, plant, and human diseases. In this study, two homolog phages (named PPAY and PPAT) that infect *Pseudomonas aeruginosa* PAO1 were isolated and characterized. The results of the phage plaque assay showed that PPAT plaques were transparent dots, while the PPAY plaques were translucent dots with a halo. Transmission electron microscopy results showed that PPAT (65 nm) and PPAY (60 nm) strains are similar in size and have an icosahedral head and a short tail. Therefore, these belong to the short-tailed phage family *Podoviridae*. One-step growth curves revealed the latent period of 20 min and burst time of 30 min for PPAT and PPAY. The burst size of PPAT (953 PFUs/infected cell) was higher than that of PPAY (457 PFUs/infected cell). Also, the adsorption rate constant of PPAT (5.97 × 10^−7^ ml/min) was higher than that of PPAY (1.32 × 10^−7^ ml/min) at 5 min. Whole-genome sequencing of phages was carried out using the Illumina HiSeq platform. The genomes of PPAT and PPAY have 54,888 and 50,154 bp, respectively. Only 17 of the 352 predicted ORFs of PPAT could be matched to homologous genes of known function. Likewise, among the 351 predicted ORFs of PPAY, only 18 ORFs could be matched to genes of established functions. Homology and evolutionary analysis indicated that PPAT and PPAY are closely related to PA11. The presence of tail fiber proteins in PPAY but not in PPAT may have contributed to the halo effect of its plaque spots. In all, PPAT and PPAY, newly discovered *P. aeruginosa* phages, showed growth inhibitory effects on bacteria and can be used for research and clinical purposes.

## Introduction

*Pseudomonas aeruginosa* (*P. aeruginosa*), a major cause of acute and chronic infections such as urinary tract infections, burn skin infections, and lung infections, is a common health problem in clinical practice (Balasubramanian et al., 2012; Tumbarello et al., 2020; John et al., 2021). It is a common food/waterborne pathogen with intrinsic antibiotic resistance causing high mortality in infected patients (Meng et al., 2020; DehghanZadeh et al., 2021). Antibiotic resistance is the major difficulty in the clinical treatment of *P. aeruginosa* infections; the current treatment methods include chemical antibiotic combination therapy (Drusano et al., 2018; Huan et al., 2019) and new drug development (Crousilles et al., 2015; Fangous et al., 2021), physical [iron control (Yu and Ma, 2017) and LED lighting control methods (Yang et al., 2020)], and biological [phage therapy (Ly-Chatain, 2014; Chegini et al., 2020)] methods. The resistant strains have emerged due to excessive antibiotic use and are more difficult to eradicate and expensive to treat (Asghar et al., 2020). The failure of antibiotics has become a global health problem (Sylvia et al., 2018). Furthermore, the continual release of antibiotic residues into the environment from hospitals, healthcare facilities, landfills, and urban wastewater treatment plants (Tuo et al., 2018) is aggravating the problem of resistant pathogens, bioaccumulation, and environmental pollution (Magrone et al., 2016). Therefore, the effective elimination of antibiotics from environmental pollution has become a worldwide concern (Huo et al., 2020). Also, with the increasing prevalence of drug-resistant strains, developing novel non-antibiotic treatments has become an urgent health issue. Phages offer significant benefits over classical antimicrobial approaches. For instance, phages can be highly targeted to certain bacteria without affecting commensal flora (Kwiatek et al., 2015), have few side effects, and are immunologically well tolerated (Roach et al., 2017). Moreover, *P. aeruginosa* phages can efficiently inactivate multidrug-resistant *P. aeruginosa* of high stability (Vieira et al., 2012), and are easy to administer (Pang et al., 2019).

Phages after infecting bacterial cells release endolysin that hydrolyzes the peptidoglycan cell wall killing bacteria (Hurley et al., 2012). Also, infecting phages include virulent and temperate phages; the virulent phages are commonly used for phage therapy for their high virulence and short lysis cycle (Townsend et al., 2021). During infection, the hollow tail of the phage can be used to transport nucleic acids (Aschalew et al., 2016). The bottom plate connected to the tail fibers at the end of the tail mainly promotes the phage integration with the bacteria (Aschalew et al., 2016). Phages can eliminate *P. aeruginosa* biofilms by disrupting the extracellular matrix and inhibiting cell population sensing activity (Chegini et al., 2020). The synergy between phage lysis and the host autoimmune defense system is named immune phage synergy requiring host neutrophils to participate in the eradication of resistant infection (Roach et al., 2017). Phage cocktails, genetically modified phages, phage lytic enzymes, and phage vaccines are the main attempts to develop phage-based treatments for bacterial infections (Zalewska-Piatek and Piatek, 2020). Phages have some therapeutic potential; for example, a wide variety of Pseudomonas phages, including *P. aeruginosa* phage Φ kz and EL, the *Pseudomonas syringae* phage φ6 (Cystoviridae), the fluorescent Pseudomonas phage OBP, etc. (Poranen et al., 2008; Matsui et al., 2017), can be used for biological, plant control, etc., so the isolation of new Pseudomonas phages remains the focus of scholars in recent years (Rombouts et al., 2016), and is mainly biased toward the study of lytic phages (Hatzopoulos et al., 2016). PSA11, LKA1, and some other *P. aeruginosa* phages (Huszczynski et al., 2019; Nordstrom et al., 2022) use LPS as an infection receptor. The polysaccharide lytic enzymes of these *P. aeruginosa* phages can reduce cytotoxicity and disrupt biofilms, and they may help to develop new narrow-spectrum antibacterial therapies (Nordstrom et al., 2022). Pseudomonas lytic phage cocktails can be used for the treatment of animals infected with *P. aeruginosa* (Flodman et al., 2019), promising to control chronic infections (Agarwal et al., 2018). Temperate Pseudomonas phage studies have also been studied, such as *P. aeruginosa* phage Pf, which was isolated from *CF* sputum and contributes to the study of biofilm mechanisms, but its effectiveness in clinical applications remains to be investigated (Burgener et al., 2019).

However, the current phage database is very inadequate limiting our knowledge of the abundance of phages (Parmar et al., 2017). Phages are highly abundant in various environments, but their interactions with bacteria are not fully understood (Hsu et al., 2019). The failure of phage therapy to achieve the desired effect is not only due to insufficient dose (Zhao et al., 2017) but may also be related to insufficient phage coverage (Criscuolo et al., 2017). Studying phage genomes can highlight the mechanisms of phage effects on the host as well as improve our understanding of genetic diversity among phages (Lood and Collin, 2011). An insufficient understanding of the phage infection mechanism of bacteria limits the choice of phage therapies (Baig et al., 2017). In the same way, there is a similar need for research on Pseudomonas phages; for example, mechanisms such as genome packaging of Pseudomonas phages need to be further explored (Niazi et al., 2020), and the current sequencing technologies for genomes such as PaP1 of *P. aeruginosa* phages still need to be improved (Hatzopoulos et al., 2016). Some phages such as Citrobacter Phage CVT22 (Tikhe et al., 2015) and *P. aeruginosa* phages PA11 have been sequenced but not yet characterized (Kwan et al., 2006). Therefore, it is essential to isolate and study novel Pseudomonas phages.

Phages, like bacteria, are abundant and widely distributed in the natural environment and play an important ecological role (Aziz et al., 2020). Phages are derived from humans and animals as well as from oceans, lakes, sediments, soils, and built environments (Al-Shayeb et al., 2020; Callanan et al., 2020). Some surveys (Mattila et al., 2015; Pires et al., 2015; Huang et al., 2018b; Edwards et al., 2019) have provided accurate information on the worldwide distribution of common phages; sewage treatment plants were found to be the most abundant resource site that can be used as a natural reservoir for obtaining phages. The isolation of new phages from the environment can greatly improve our understanding of phage-bacterial interactions potentially providing biological options for the bacteria resistance problem (Jiang and Zheng, 2020). Accordingly, to develop efficient phages targeted for the treatment of *P. aeruginosa*, we isolated two newly isolated phages, named PPAY and PPAT, from wastewater treatment plants and studied their biological and genomic characteristics. This study increases our knowledge of the diversity of *P. aeruginosa* phages, providing a resource for the control of *P. aeruginosa* infections.

## Materials and methods

The host bacterial strain of phages was a wild-type *P. aeruginosa* strain PAO1 (ATCC15692). Selective agar medium (Pseudomonas CN Agar, Qingdao Hope Bio-technology Co., Ltd.) and LB liquid medium were used for the identification and amplification of *P. aeruginosa,* respectively. The strains were screened on the selective medium plates, and then single clones were inoculated in LB liquid medium for overnight at 37°C. The PAO1 culture was used for further experiments including phage isolation, proliferation, counting, and so on.

### Sampling locations and sample collection methods

The activated sludge samples were collected from a municipal wastewater treatment plant located in Tianjin, China. The samples were packed in 500 ml plastic bottles, sealed, and placed in a foam box with ice at 0–4°C. The samples were processed as soon as possible after reaching the laboratory. The samples were first mixed well with an equal amount of Pseudomonas isolation broth medium (PIB, Qingdao Hope Bio-technology Co., Ltd.) and then incubated at 37°C for 24 h at 150 rpm. Next, the culture mixture was added with a final concentration of 5 mmol/l sodium pyrophosphate and left at room temperature for 30 min under the dark to dissociate the phages adsorbed on the activated sludge (Pan et al., 2019). After ultrasonication for 1 min using an ultrasonic cleaner (KQ-100E, 47 kHz, Kunshan Ultrasonic Instruments Co., Ltd.), the sludge was centrifuged at 10,000 × *g* for 5 min to collect the supernatant. Next, smaller contaminants were removed by filtration using a 0.22-μm filter membrane (Millipore; Park et al., 2012), and the filtrate was stored at 4°C.

### Phage isolation and purification

The traditional double-layer agar protocol was used to determine the number and activity of phages (Topka et al., 2019). After mixing the phage filtrate (obtained in section 2.2) with host cells PAO1 for 24 h, the bacteria cells were lysed with chloroform. The culture was then centrifuged (6,000 × *g*, 10 min, 4°C) to collect the supernatant, which was filtered through a 0.22 μm nitrocellulose filter to remove fine material (Nagayoshi et al., 2016). The filtrate was mixed with log-phase PAO1 cells and then used for plaque assay using the double-layer agar plate method (Hsu et al., 2019). The individual different pattern plaques were picked for further purification. Each pattern plaque was purified more than thrice until the plaque of single morphology was obtained. The purified phage particles were amplified and stored in the PIB culture medium containing 20% (v/v) glycerol at −80°C (Li et al., 2019).

The purified phage particles were inoculated into host bacteria PAO1, and incubation was carried out for 24 h at 37°C and 150 rpm. Next, the bacteria were lysed with chloroform, and bacterial debris was removed by centrifugation and filtration (0.22 μm microporous membrane). The phage was separated by ultrafiltration (Millipore, 10 K da, centrifuged at 3,000 × *g* for 1 h). After three times centrifugal washing with sterilized ultrapure water, the phage on the ultrafilter membrane was recovered in 1 ml of sterilized ultrapure water. Phage titers were calculated using the double-layer agar plate method. The phage was stored at 4°C for further experiments.

Host range was determined by a spot titration protocol using seven *P. aeruginosa* isolates (*P. aeruginosa* PAO1 ATCC 15692, *P. aeruginosa* ATCC 9027, *P. aeruginosa* cmccb 10,101, *P. aeruginosa cmccb* 10,102, *P. aeruginosa* cmccb 10,211, *P. aeruginosa* ATCC 15442, and *P. aeruginosa* ATCC 27853).

### One-step growth curves

The one-step growth curve determination method was adapted from Kropinski (2018). Briefly, 1 ml exponential phase host strain culture in the PIB medium (cell density ~ 10^7^ cfu/ml) was infected with 10 μl of phage suspension (titer 2 × 10^6^ pfu/mL) to reach an MOI (multiplicity of infection) of 0.2; the mixture was incubated for 5 min at 37°C. Next, the mixture was centrifuged at 10,000 × *g* for 1 min to remove unadsorbed phages. The sediment was washed with PBS and then re-suspended in 1 ml PBS. Next, 0.1 ml bacterial suspension was added to 30 ml of fresh liquid LB medium and incubated at 37°C and 150 rpm. One mL samples were taken out every 10 min for the first 2 h and then every 30 min for the second 2 h. At the end of sample collection, the culture was added with 20 μl of chloroform with vigorous shaking at 3000 r/min for 20 min and then centrifuged at 10,000 × *g* for 1 min. The titer of the supernatant was estimated using the double-layer agar method. All experiments were conducted with three replicates. The *t_n_*/t_0_ was used as the normalized result, where *t*_0_ and *t_n_* are the phage titers at time 0 and *n* min, respectively.

### Adsorption rate constant

The adsorption constants were expressed as volume/time (mL/min) and were measured as described by Abedon et al. (Kropinski and Clokie, 2009). A log-medium bacterial culture was diluted to OD_600_ of 0.1–0.2. Then, 1 ml of phage stock (2 × 10^5^ PFU/mL) was mixed with 9 ml of bacteria suspension (MOI = 0.001) with swirling. The mixture was cultured at 37°C. Next, 50 μl samples were removed from the cell suspension mixture every 1 min and centrifuged at 10,000 × *g* for 30 s to pellet the bacteria. The obtained supernatant was diluted with PBS to measure the phage titer using the double-layer agar method (Koonjan et al., 2020). The assay was performed in three independent experiments. The adsorption rate constant was determined as per the Equation (1).


(1) $$k=-\ln\left(P/P_{0}\right)/Nt$$

where *k* is the adsorption rate constant, *P*_0_ and *P* are the starting and ending phage titers, respectively, *N* is the bacterial density, and *t* is the time (min) when adsorption occurred.

### Transmission electron microscopy

A single drop of phage stock was placed onto the 200-mesh copper grid covered with Formvar/carbon film (Beijing Zhongjingkeyi Technology Co., Ltd.) for 10 min at room temperature. The excess solution was aspirated with filter paper, and then one drop of 2% potassium phosphotungstate (pH 7.0) was placed on the surface of the copper grid for 2 min for staining. The access staining solution was aspirated with filter paper and dried naturally. The morphology of phages was observed using a Jem2100 transmission electron microscope (JEOL, Tokyo, Japan) at 80 kV.

### Phage sequencing and genome analysis

Fresh phage stock solution (prepared in Section “One-Step Growth Curves”) was passed through a DNA adsorption column (Solarbio) for 10 min to remove residual nucleic acid fragments. Next, the column containing phage stock was centrifuged at 10,000 × *g* for 1 min to collect the filtrate. The adsorption procedure was repeated one more time, and the filtrate was obtained after centrifugation. Furthermore, any residual DNA was removed by DNase I. Finally, the filtrate sample was analyzed by gel electrophoresis to ensure the complete removal of residual DNA. Phage DNA was extracted using the DNA viral genome extraction kit (Solarbio, China) following the manufacturer’s instructions. The purity and integrity of DNA were analyzed by agarose gel electrophoresis and DNA concentration was determined by NanoDrop One (Thermo Fisher Scientific 5225, United States). The genomic DNA of both the phages was purified and sequenced on the Illumina platform (Allwegene Tech., Beijing, China). Briefly, the DNA in samples was broken randomly into fragments of ~300 bp by Covaris ultrasonic fragmentation, and the whole database was generated by terminal repair, clarification, and PCR extension. After library construction, initial quantification was performed with Qubit 2.0. The library was diluted to 2 ng/μL, and the addition gap size in the library was checked by Agilent 2,100. After the libraries passed the quality assays, sequencing was performed with Illumina HiSeq. The intensity and amount of phage downstream data determines whether the next step of the study could be carried out. A 300 bp database was prepared for each phage and quality control was performed by Trimmomatic (v0.36) program. Before segment assembly, intermediate high-quality sequencing regions were selected and K-mer of a specific width was used one by one for genome size assessment. Each K-mer was counted as a percentage of the 31 K-mer depths and the frequency of each depth was calculated. The sequencing data were assembled with SPAdes (v3.13.0) software.

Prokka (1.13.7) software was used for annotation prediction of the phage genome and RepeatMasker (v4.0.9) software was used for repetitive sequence analysis. Protein annotation was performed according to common functional databases non-redundant (NR), Swiss-Prot, Gene Ontology (GO), Kyoto Encyclopedia of Genes and Genomes (KEGG), Cluster of Orthologous Groups (COG), InterProScan, carbohydrate-active enzymes (CAZymes), etc.

The annotated phage sequences of PPAY (accession number MZ727202) and PPAT (accession number MZ727332) have been deposited at NCBI-GenBank. The gene clusters of the predicted functional proteins of PPAT and PPAY were mapped using the chiplot tool (https://www.chiplot.online/gene_cluster.html). Genomes of PPAT and PPAY were compared by mauve software (D'Auria et al., 2010).

### Characterization of phage structural proteins

Purified phage samples (1 × 10^10^ PFU/mL) were mixed with 5× sample loading buffer and boiled for 10 min before analysis by 12% SDS-PAGE (Fortier et al., 2006; Uchiyama et al., 2014).

### Comparative genome analysis

For kinship analysis, genomic sequences with certain evolutionary relationships were acquired from the GenBank database on June 15th, 2022, and compared using the Clustal W program. A neighbor-joining phylogenetic tree was constructed using the MEGA X software with 3,000 bootstrap replications (Pacífico et al., 2019).

### Phage inhibition of PAO1

To assess the possible inhibit effect of PPAT and PPAY on the growth of PAO1, a growth curve was constructed (Wang et al., 2016). The approximate number of bacteria was estimated based on the density of bacteria culture by assessing the bacterial growth period using OD_600_ (Wang et al., 2016). PAO1 bacteria were divided into three treatment groups, one with LB, one with PPAT, and one with PPAY. Each group was set up in three replicates in a 100-well plate at 200 μl per well. Sample OD_600_ was measured every 1 h for 18 h using a fully automated growth curve analyzer (BIOSCREEN C^0^ PRO).

### Screening and identification of phage-resistant mutants

The previous method was modified as follows (Guglielmotti et al., 2007; Tomat et al., 2013). After PPAT and PPAY were infected with bacteria separately for 3 days (MOI about 100), the surviving bacteria were cultured up again, and CN solid medium was cultured up as a putative stock of phage-resistant bacteria after three successive scribing and purification. The phage was added into PAO1 and its phage-resistant strain, respectively (MOI about 100), and after 24 h of incubation, the surviving bacteria were taken and cultured up again with phage, until the resistant strain was clearly distinguished from the control group and grew up rapidly. The resistant strains that survived for the 3rd time were identified as phage-resistant strains after three further passages of culture under the same conditions (Supplementary Figure S1). Each group was set up with phage-infected wild type as the control group.

## Results

### Phage morphology

**Figure 1:**
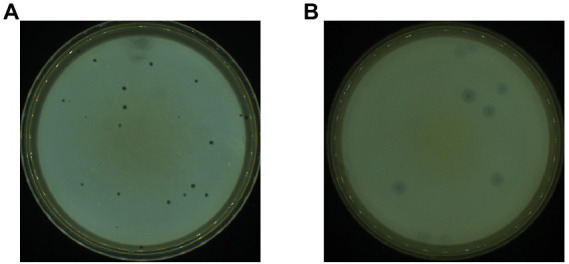
Plaque morphology. **(A)** PPAT and **(B)** PPAY.

Based on the double-layer plate method, two strains of phage-infected *P. aeruginosa* PAO1 were isolated from sludge samples collected from the Wastewater Treatment Plant after several rounds of separation and purification. These two strains showed completely different plaque morphologies and were named PPAT and PPAY, respectively. The plaque morphology of PPAT (Figure 1A) was a clear regular dot of 1–2 mm in diameter, while PPAY plaque was a ring-shaped area with an obvious circular translucent hole in the center and a large ring-like aperture at the periphery that continued to grow up to 2 cm in 24 h (Figure 1B).

Host range tests (Supplementary Table S1) showed that the two newly discovered phages only infected PAO1, indicating that the lysed range of phages was very narrow, and the two phages have a specific choice for the host. However, there are also phages that can lyse a variety of hosts, such as *P. aeruginosa* PHAge ZC01, which can lyse not only PA14 but also *P. aeruginosa* 5757, H6044, and H6086 (Amgarten et al., 2017).

**Figure 2:**
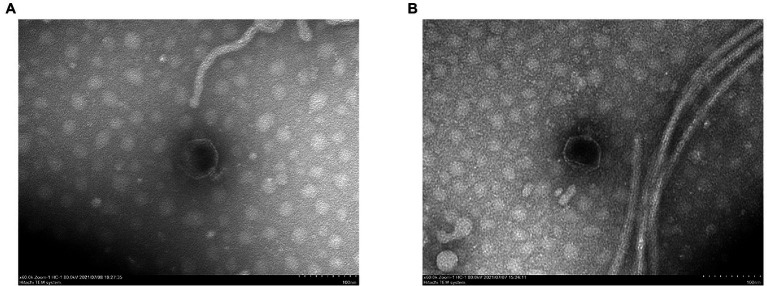
Transmission electron micrographs showing the virion particle morphology. **(A)** PPAT and **(B)** PPAY. Scale bar: 100 nm.

After staining, the structural morphology of the isolated phages was observed by TEM. The PPAT phage structure exhibited an icosahedral head of ~65 nm diameter and a short non-contractile tail of ~20 nm (Figure 2A). The structure of the PPAY phage was similar to that of PPAT showing an icosahedral head of ~60 nm and a short non-contractile tail of ~20 nm (Figure 2B). Both the phases were designated to the *Podoviridae* family following the criteria of Ackermann (2007).

### Infection characteristics of phages

**Figure 3:**
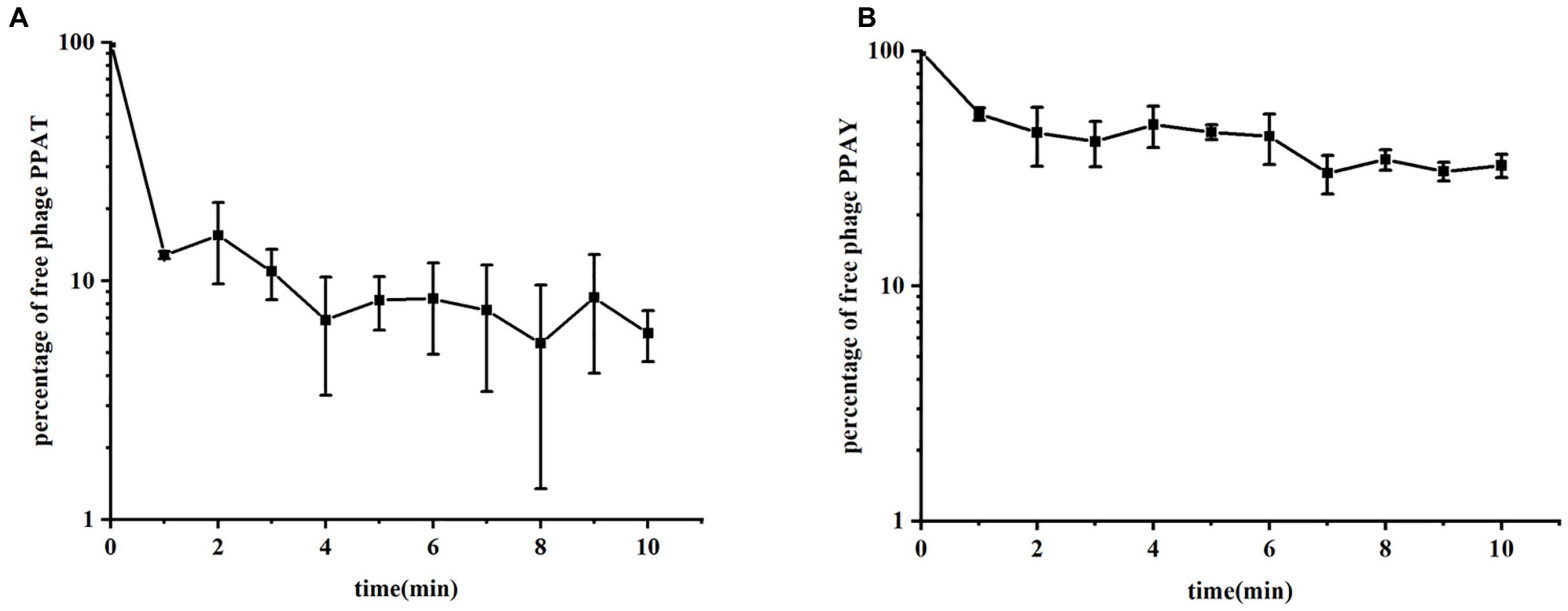
The phase adsorption curves. **(A)** PPAT and **(B)** PPAY. The adsorption curve was drawn with time as abscissa and the percentage of free phage as ordinate. Error bars indicate standard deviations of biological triplicates.

The adsorption rate constant, latency period, and burst size are the fundamental characteristic features of bacterial phage infection (Weber-Dąbrowska et al., 2016). We established the adsorption curves to compare the adsorption efficiency of phages to PAO1 strains (Liu et al., 2018). The results showed that the phase adsorption rates showed significant variations over time, which could be related to the number of infected bacteria or relevant environmental factors (Gibson et al., 2019). The respective adsorption curves PPAT and PPAY at 37°C are shown in Figure 3. The adsorption experiment lasted for 10 min, and the adsorption rate constants were calculated as in Hyman and Abedon (2009). The results showed that at an MOI of 0.001, the adsorption rate constants of PPAT and PPAY were about 5.97 × 10^−7^ and 1.32 × 10^−7^ ml/min at 5 min, respectively. PPAT showed a relatively fast absorption rate. Previous studies showed that a higher adsorption rate of phage particles increases the lysis rate (Nguyen and Kang, 2014). Therefore, PPAT should more rapidly lyse the bacteria.

**Figure 4:**
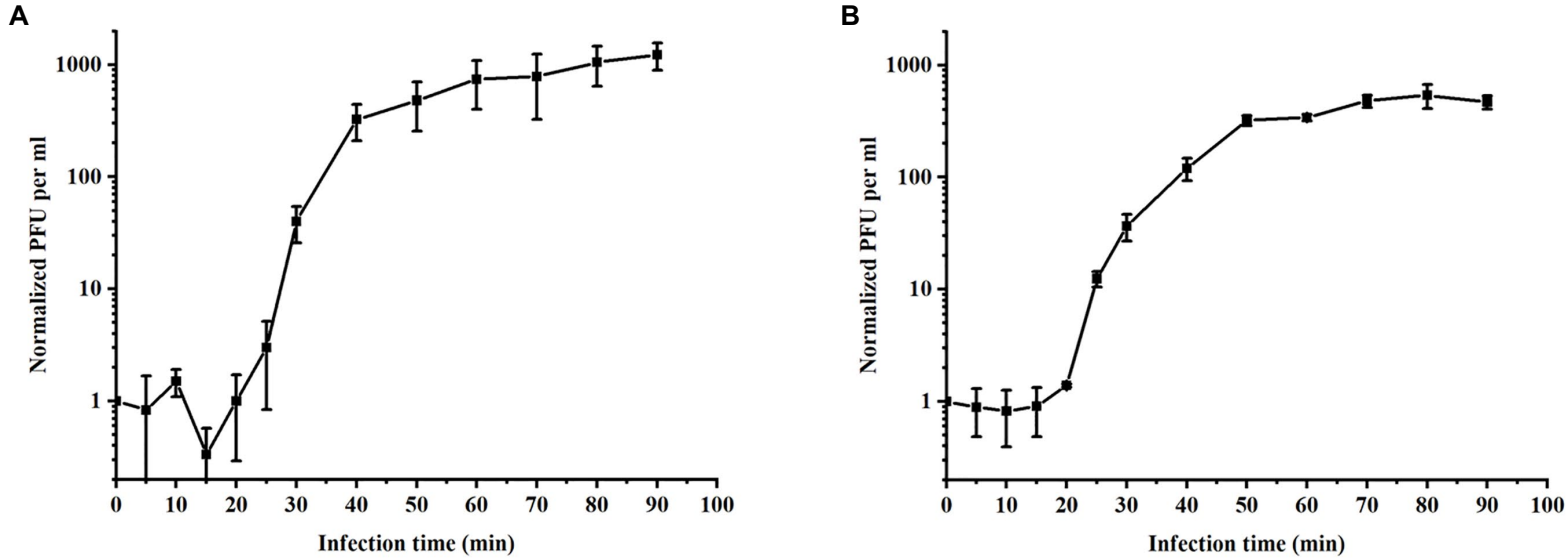
One-step growth curves of phages. **(A)** PPAT and **(B)** PPAY. Error bars indicate standard deviations of biological triplicates.

The growth parameters of phages were determined by one-step growth curve experiment (Imam et al., 2019), reflecting the dynamic changes in the number of particles during phage replication (Li et al., 2019). One-step growth curves at 37°C were plotted to ascertain the latency and burst size of PPAT and PPAY. The phage burst size denotes the mean phage titer value in the plateau phase as a ratio to the titer value in the latent phase (Lee et al., 2019). PPAT with a lag time of 20 min (MOI = 0.2), a burst time of 30 min, and a burst size of 953 PFUs/infected cell, the secondary outbreak reached the steady-state population after ~120 min (Figure 4). PPAY with a lag time of 20 min (MOI = 0.2), a burst time of 30 min, and a burst size of 457 PFUs/infected cell, the secondary outbreak reached the steady-state population after ~120 min (Figure 4). These results are consistent with previous studies (López-Cuevas et al., 2011; Huang et al., 2018a), in which secondary adsorption began after 90 min, indicating that non-adsorbed cells were still present after the first burst, even if the MOI was ≥1.

### SDS-PAGE analysis of phage structural proteins

**Figure 5:**
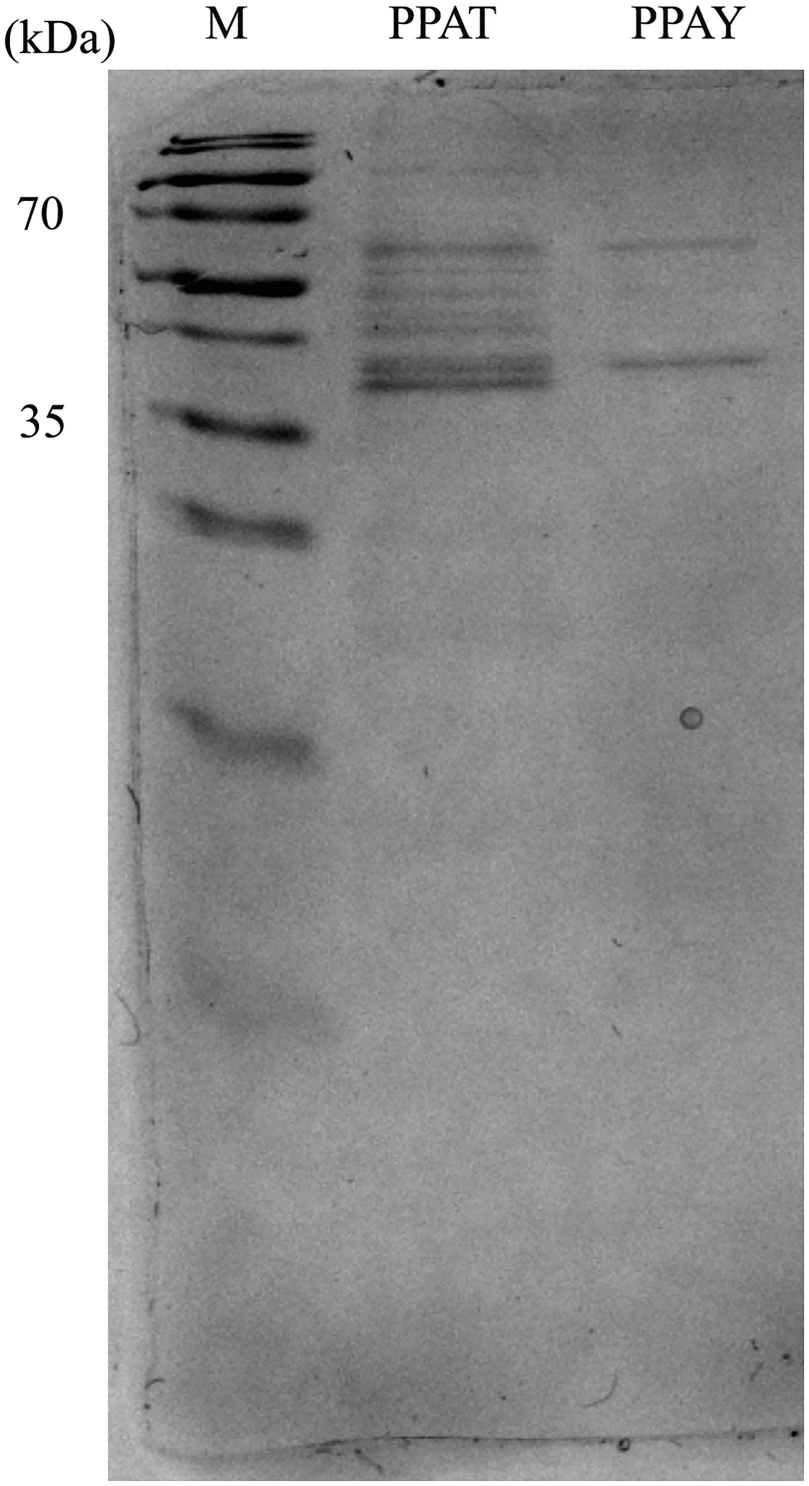
SDS-PAGE analysis of phage proteins.

Based on the optimization of phage therapy without the effect of endotoxins, the structural proteins of phages were analyzed by SDS-PAGE to gain insight into the protein profile of phages (Jia et al., 2020). The results showed that each phage contained a minimum of 10 structural proteins (Figure 5). The size of the most abundant structural protein is 35–70 kDa. The major structural proteins (Ahamed et al., 2019) in Figure 5 include phage terminase (large subunit), phage portal (connector) protein, and some hypothetical proteins. The SDS-PAGE analysis can only reveal limited knowledge, full genome study is required to better learn the role of all phase proteins (Lu and Breidt, 2015). According to genome annotation results, the phages are mainly composed of phage tail protein, bacteriophage head-to-tail connecting protein, major coat protein, and other structural proteins. Also, the main enzymes related to phage reproduction and metabolism such as DNA helicase and DNA polymerase are present (Lu et al., 2013). The proteins such as tail fiber protein, head-tail linker protein, and hydrolase are critical and fundamental to the life span of phages. It is suggested that phages obtained genes related to host infestation during the co-evolutionary process (Yuan and Gao, 2016; Tavares, 2018).

### Genomic features of phages

The whole genomes of PPAT and PPAY against *P. aeruginosa* PAO1 were sequenced by Illumina HiSeq and the assembly was completed by SPAdes (v3.13.0). The genomes of both phages were linear double-stranded DNA. The genome size of PPAT is 54,888 bp with 45.15% GC content, while the PPAY genome size is 50,154 bp with 44.51% GC content. The putative ORFs in the genome of phage PPAT were predicted after genome annotation. About half of ORFs (179 of 352; 51%) were located on the positive strand and the other 173 ORFs (49%) were on the negative strand. Among the predicted ORFs, some belonged to structurally and functionally related genes already published or validated in databases, while the others belonged to the unknown category of the hypothetical phage proteins, i.e., solitary proteins (Table 1). The PPAT genome was divided into four main modules, which complemented each other constituting the complete phage life cycle. For example, ORF123 is involved in the lysis module. The proteins encoded by ORF218 and ORF336 were DNA primase or helicase, which might engage in phage DNA replication. Meanwhile, ORF105 encodes the large subunit of phage terminator enzyme, which is potentially associated with phage DNA packaging together with the ORF106 encoding portal protein. The phage portal protein functions as a junction channel to facilitate the DNA entry into the bacterium for genome assembly (Sun et al., 2015). The structure module includes ORF50 and ORF13. ORF105 shares 28.26% identity with the large subunit of terminase enzyme, which assists in DNA translocation and packaging termination (Duffy and Feiss, 2002). The PPAT genome encodes a homolog (ORF340) of thymidylate synthase ThyX (EC 2.1.1.148) which is an essential prokaryotic enzyme (Myllykallio et al., 2018), also a NAD(P)H oxidase that uses flavin adenine nucleotide to mediate hydride transfer (Leduc et al., 2007), and an essential precursor for DNA synthesis (Takezawa et al., 2011).

**Table 1:** Predicted ORFs of phage PPAT.

ORFs	Strand	Frame	Start	End	Length (bp)	Molecular mass (kD)	Encoded protein
13	+	1	6,715	6,993	279	10.23	Phage capsid and scaffold
50	+	1	44,158	44,424	267	9.79	Phage capsid and scaffold
56	+	2	4,004	5,782	1,779	65.23	Streptococcal hemagglutinin protein
105	+	2	38,297	39,904	1,608	58.96	Phage terminase, large subunit
106	+	2	39,917	41,671	1,755	64.35	Phage portal (connector) protein
123	+	3	5,769	6,206	438	16.06	Phage lysozyme R (EC 3.2.1.17)
218	−	1	20,511	18,796	1,716	62.92	DNA primase/helicase, phage-associated
225	−	1	12,963	12,196	768	28.16	Phage exonuclease
229	−	1	11,070	10,309	762	27.94	Predicted ATPase related to phosphate starvation-inducible protein PhoH
230	−	1	9,966	8,998	969	35.53	Ribonucleotide reductase of class Ia (aerobic), beta-subunit (EC 1.17.4.1)
275	−	2	13,622	13,197	426	15.62	Phage-associated homing endonuclease
320	−	3	26,179	24,848	1,332	48.84	Glutamine amidotransferase, type II
322	−	3	23,431	22,475	957	35.09	Amidoligase
336	−	3	14,494	13,622	873	32.01	DNA polymerase I (EC 2.7.7.7)
340	−	3	11,623	10,949	675	24.75	Thymidylate synthase ThyX (EC 2.1.1.148)
341	−	3	10,198	9,950	249	9.13	Phage thioredoxin or glutaredoxin (Nrd; ACLAME 238)
342	−	3	8,929	7,310	1,620	59.4	Ribonucleotide reductase of class Ia (aerobic), alpha-subunit (EC 1.17.4.1)

Likewise, the putative ORFs in the PPAY genome were predicted. Half of the putative ORFs (179 of 351; 51%) were situated on the positive strand and the other 172 ORFs (49%) were on the negative strand. Functional annotation of the putative ORFs of PPAY is shown in Table 2. ORF310 is part of the lysis module. ORF32 and ORF79 participate in phage genome reproduction, while ORF293 together with ORF294 might engage in phage genome packaging. The structure module includes ORF202, ORF239, and ORF244. Also, one putative tRNA participating in the genomic transcription in the infested bacteria was identified. The presence of tRNAs in the phage genomes suggests that phages are less dependent on their hosts for protein synthesis involving host tRNAs (Saks and Conery, 2007).

**Table 2:** Predicted ORFs of phage PPAY.

ORFs	Strand	Frame	Start	End	Length (bp)	Molecular mass (kD)	Encoded protein
16	+	1	10,570	11,901	1,332	48.84	Glutamine amidotransferase, type II
18	+	1	13,318	14,274	957	35.09	Amidoligase
32	+	1	22,255	23,127	873	32.01	DNA polymerase I (EC 2.7.7.7)
36	+	1	25,126	25,800	675	24.75	Thymidylate synthase ThyX (EC 2.1.1.148)
37	+	1	26,551	26,799	249	8.47	Phage thioredoxin or glutaredoxin (Nrd; ACLAME 238)
38	+	1	27,820	29,439	1,620	59.4	Ribonucleotide reductase of class Ia (aerobic), alpha-subunit (EC 1.17.4.1)
79	+	2	16,238	17,953	1,716	62.92	DNA primase/helicase, phage-associated
86	+	2	23,786	24,553	768	28.16	Phage exonuclease
90	+	2	25,679	26,440	762	23.76	Predicted ATPase related to phosphate starvation-inducible protein PhoH
91	+	2	26,783	27,751	969	35.53	Ribonucleotide reductase of class Ia (aerobic), beta-subunit (EC 1.17.4.1)
142	+	3	23,127	23,552	426	15.62	Phage-associated homing endonuclease
202	−	1	30,034	29,756	279	10.23	Phage capsid and scaffold
239	−	2	49,812	48,895	918	33.66	Phage tail fiber (ACLAME 1074)
244	−	2	42,489	42,223	267	9.79	Phage capsid and scaffold
250	−	2	32,745	30,967	1,779	65.23	Streptococcal hemagglutinin protein
293	−	3	48,350	46,743	1,608	58.96	Phage terminase, large subunit
294	−	3	46,730	44,976	1,755	64.35	Phage portal (connector) protein
310	−	3	30,980	30,543	438	16.06	Phage lysozyme R (EC 3.2.1.17)

**Figure 6:**
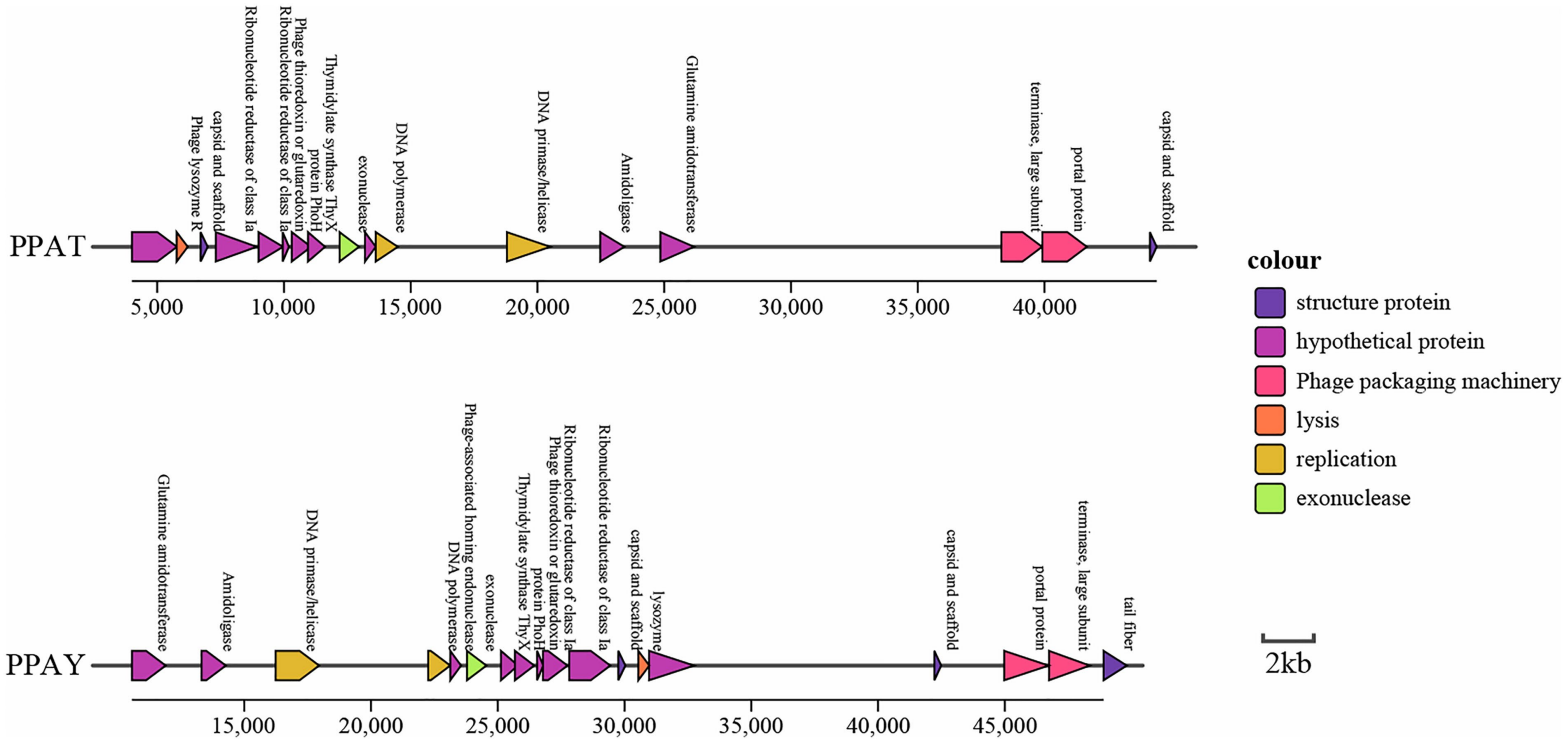
Cluster of phage genes. Genes with the same color denote homologs.

Concerning the functions of predicted genes, ORF239 in the PPAY genome is homologous to a known phage tail fiber gene. ORF239 also present in PPAT (Sandstedt et al., 2010). Previous studies showed that phage depolymerase is a portion of the tail fiber (Chen et al., 2018). Therefore, we speculated that a small portion of the tail fibers of PPAY, the protein encoded by ORF239, could be associated with depolymerase. The ORFs forming the cluster of phage genes are shown in Figure 6.

**Figure 7:**
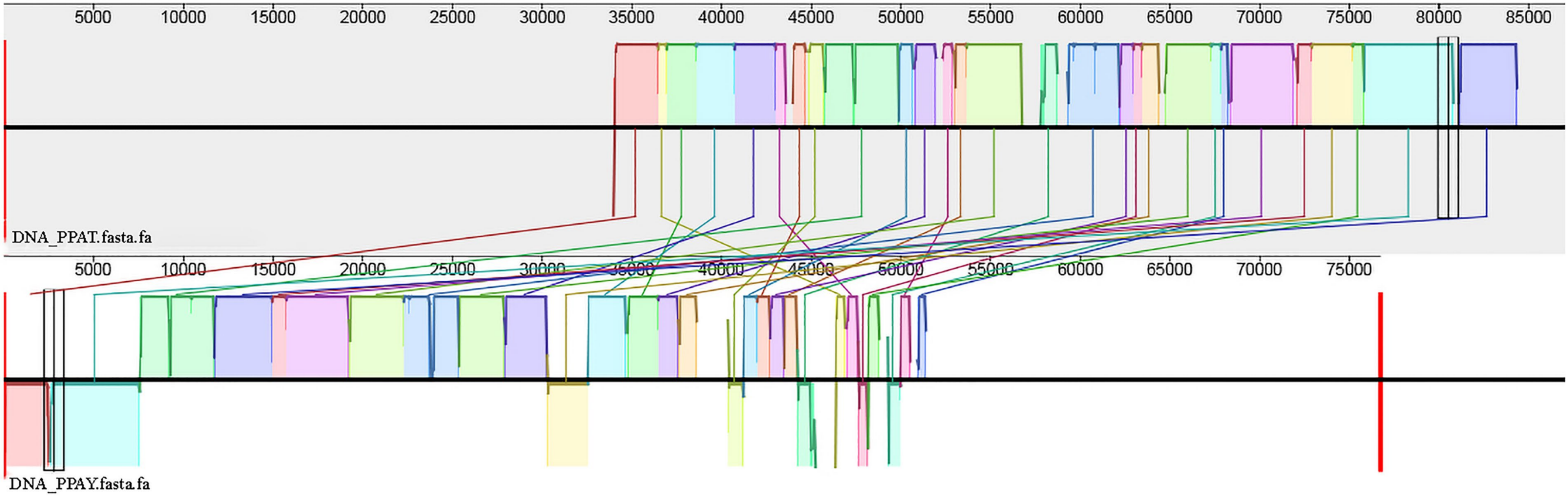
Genomic collinearity between PPAT and PPAY.

PPAT is very related to PPAY (99% coverage and 100% identity). The collinear relationship between the two genomes also confirms the same (Figure 7).

**Figure 8:**
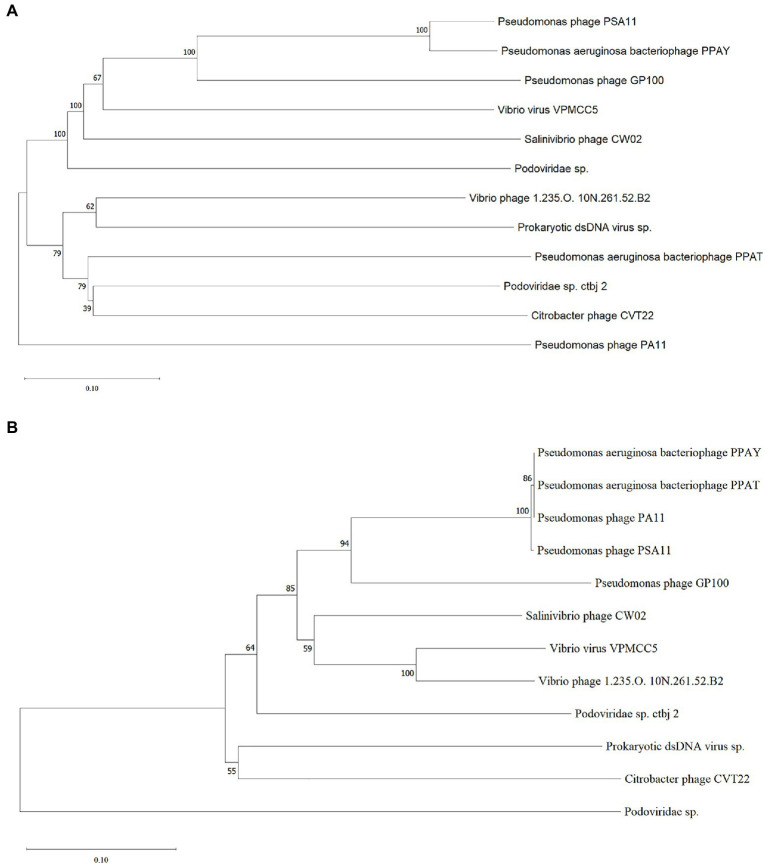
The phylogenetic tree of PPAT and PPAY. **(A)** Phylogenetic tree of PPAT and PPAY genomes, related sequences of PPAT and PPAY were obtained from GenBank and the neighbor-joining phylogenetic tree was constructed by Mega X with 3,000 bootstrap replicates. **(B)** A phylogenetic tree was generated using the sequences of the large terminase subunit. The proteins showing relatedness with large terminase subunit in PPAT and PPAY were obtained from GenBank and the neighbor-joining phylogenetic tree was constructed by Mega X with 3,000 bootstrap replicates.

To investigate the relationship between the newly isolated phages and other phages, we performed a blastn analysis of the entire genomes of PPAT and PPAY with the NCBI nucleotide collection database. We found that the PPAY and PPAT genomes are related. Furthermore, the phylogenetic tree shown in Figure 8 indicated that *P. aeruginosa* phage PSA11, *Pseudomonas* phage PA11, PPAT, and PPAY are closely related. From the perspective of genome evolution tree, PPAT and PPAY were in the same major branch and have certain evolutionary differences.

### Phage inhibition effect on the bacterial growth and phage-resistant mutants

**Figure 9:**
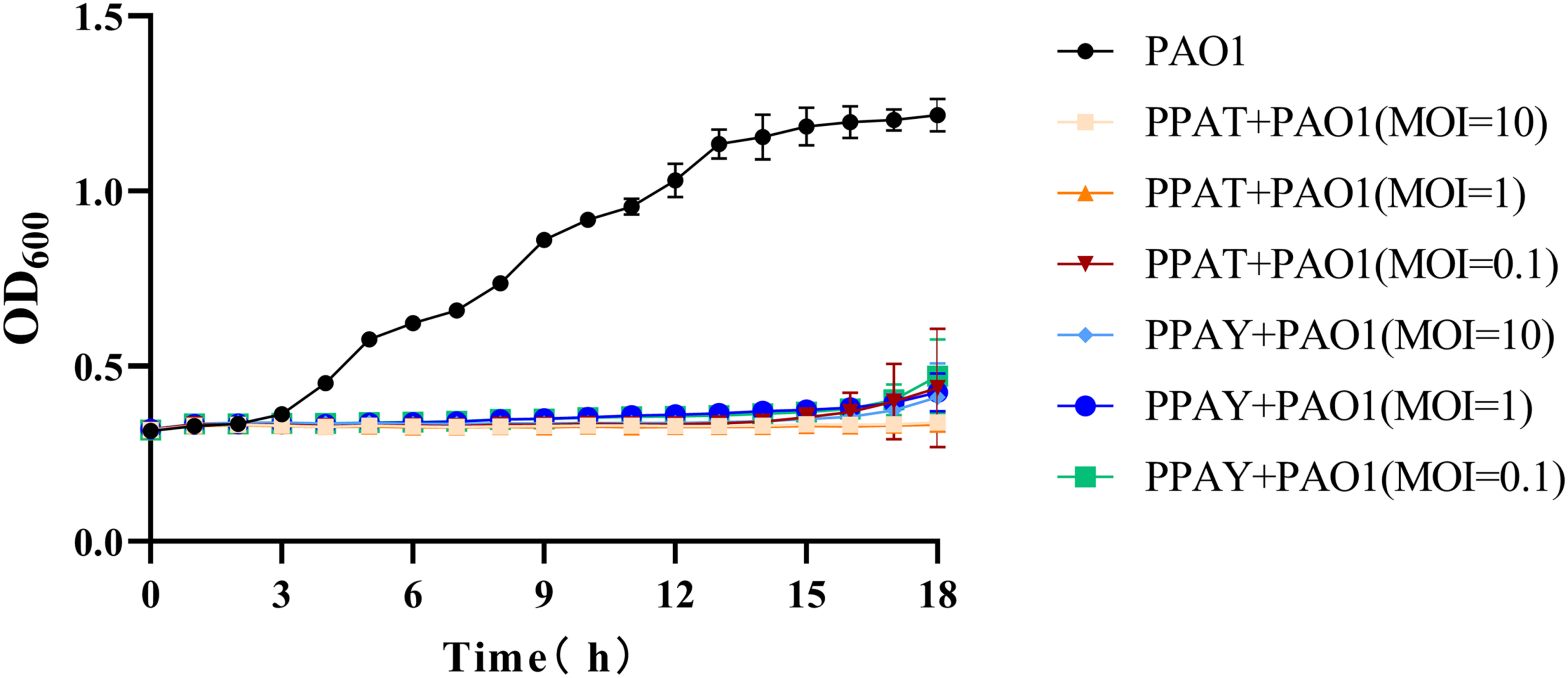
Phage infection inhibits bacterial growth.

The test results showed that the normal PAO1 strain grew rapidly after 2 h (Figure 9). The PAO1 strain infected with PPAT phage showed no growth up to18 h, while the PAO1 strain infected with PPAY grew up after 15 h with different MOI. This suggests that the growth inhibition effect of PPAT was better than PPAY. Also, a higher MOI of PPAY and slower growth of PAO1 indicated the better growth-inhibiting effect of PPAY.

After the addition of phages (MOI = 100), the growth trends of PAO1 bacteria PAO1ppat (PPAT-phage-resistant mutant) and PAO1ppay (PPAY-phage-resistant mutant) were similar to those of the wild type without the addition of phages within 18 h. Moreover, PPAT and PPAY phage were able to inhibit wild-type PAO1 for about 10 h (4–14 h), but the phage-resistant strains grew rapidly at 4 h. The effect of phage inhibition on resistant strains was significantly weaker. In contrast, *P. aeruginosa* phage K5 (MOI = 1) had some inhibitory effect on phage-resistant strains 1–9 (You et al., 2018).

## Discussion

Phages are highly host-specific, safe, and ubiquitous in the environment and do not harm humans (Rohwer and Edwards, 2002; Atterbury, 2009). There is a growing demand for phages in several fields, including human and veterinary medicine, biologics, food supply, and cosmetics (Luong et al., 2020). *P. aeruginosa,* a Gram-negative, widespread, multidrug-resistant pathogen causes complex diseases that are difficult to cure (Azam and Khan, 2019). In recent years, phages have received increasing attention, and are believed to replace antibiotics as a solution to antibiotic resistance in bacteria (Yang et al., 2021). Previous studies have shown that phage infection inhibits bacterial growth, which increases with increasing MOI (Imam et al., 2019). At MOI of 0.1 or 1, PPAT and PPAY were more effective than phage MIJ3 in inhibiting bacteria (Imam et al., 2019). Phages are promising methods for controlling infections with normal bacteria as well as drug-resistant bacteria. The main advantages of phages are that they are very specific to the host, can evolve with the host, are self-limiting, and do not cause side effects (Dedrick et al., 2019).

Phages can be readily obtained from seawater or sewage. Phages IME-JL8 (Jia et al., 2020), PVP-SE1 (Wongsuntornpoj et al., 2014), phiYeO3-12 (Hamdi et al., 2016), etc. were isolated from sewage, indicating that sewage is a natural reservoir of phages. In this study, two *P. aeruginosa* PAO1 lytic phages were isolated from sewage treatment plants, named PPAT and PPAY. PPAT is similar to the virulent phages infecting *P. aeruginosa* PA14 and exhibits as a plaque of about 2 mm in diameter (Amgarten et al., 2017). PPAY is similar to phage BF25/12 of *Podoviridae* family exhibiting halo of plaques 6 mm in diameter (Alič et al., 2017). Morphologically, these belong to the *Podoviridae* family and possess an icosahedral head and a very short tail, which is almost invisible. Similarly, a previously reported phage PD-6A3 has a head size of ~50 nm, a tail of ~10 nm, and belongs to the *Podoviridae* family (Wu et al., 2019). PPAT and PPAY were similar to the morphology of the phages of *Podoviridae* found in Alič (Alič et al., 2017), with a head size of about 55 nm and a tail of about 14 nm. The PPAT and PPAY phages share high relatedness between their genomes and are homologous to Pseudomonas phage PA11 (Kwan et al., 2006). However, the gene encoding the tail fiber protein was not predicted in PPAT; instead, PPAY contained the phage tail fiber protein (ACLAME 1074). The morphological differences in the tail structure between PPAT and PPAY can be attributed to small variations in their genome (Thanh et al., 2020).

In addition, the tail fibers may affect the release of polysaccharide depolymerase. Polysaccharide depolymerase, an important phage protein that degrades polysaccharides or extracellular polymeric substance (EPS), was first discovered in 1956; this enzyme is responsible for the classic glow-like appearance around plaque spots (Latka et al., 2017). The absence of tail fiber protein-encoding genes in PPAT but presence in PPAY suggests that PPAY is more likely to produce tail fiber proteins and thus may contain more depolymerase (Chen et al., 2018). This explains the large center spot in the plaque of PPAY.

The previous study said that about 50% of *P. aeruginosa* phage MIJ3 attached to PAO1 within 6 min (Imam et al., 2019), while about 90% of PPAT quickly adsorbed to PAO1 within 1 min, and about 50% of PPAY adsorbed to PAO1 within 1 min, so the adsorption rate of the two newly discovered phage strains was higher than that of phage MIJ3. Concerning the adsorption rates, the adsorption rate of PPAT was larger than that of PPAY within the first 5 min of the assay. Previous studies showed that the phage with a lower adsorption rate produces a larger plaque area (Gallet et al., 2012). This is consistent with the fact that the plaque area of PPAT was smaller than that of PPAY. The tail structure of the phage determines its specific recognition and adsorption process to the host (Fan et al., 2019). Interestingly, there could be some differences in the tail structure between PPAT and PPAY.

Concerning the one-step growth curve assays, the incubation period (20 min) and outbreak time (30 min) of PPAT and PPAY are similar to previous reports. For instance, a previously reported phage Vp670 of the *Podoviridae* family had a latent period of 30 min, which subsequently showed a continuous sharp increase in phage titers lasting 30 min. The burst size of phage PPAT (953 PFUs/infected cell) was larger than PPAY (457 PFUs/infected cell). These two newly identified phage strains had a shorter latency time and a larger burst size than MIJ3 (45–50 min, 68 PFUs/infected cell; Imam et al., 2019). The thioredoxin protein (Zhang et al., 2009) of PPAT and PPAY is necessary for their binding and propagation and is related to burst size. Upon long enough growth of ~100 min, the number of secondary outbreaks or multiple outbreaks increases during the growth of PPAT and PPAY. This is mainly due to the presence of uninfected hosts after the first outbreak. Large burst volume is a key characteristic of effective antimicrobial phage. In addition, as an antimicrobial agent, phages with large bursts indicate advantages of selection. The phage with large bursts allows increasing the initial dose by a few hundred times in a short period (Xu et al., 2018). Therefore, PPAT, with a large burst size, has more potential to be used for large-scale biological control of bacterial pathogens than PPAY.

Phage PPAT and PPAY are virulent phages that are phylogenetically related to *P. aeruginosa* bacteriophage PA11 and have 18 and 53 genes coding for putative function and unknown function, respectively. The *P. aeruginosa* phages TC6, O4, and IME180 showed significant relatedness and are very phylogenetically close to PA11. Therefore, these were classified as a new genus of phages infecting *P. aeruginosa* named *Pa11virus* (Tang et al., 2018). Similarly, classifying PPAT and PPAY as *Pa11virus* contributed to the diversity and classification of *Podoviridae*. During ORF prediction, streptococcal hemagglutinin protein (HA) was predicted in both PPAT (ORF56) and PPAY (ORF250). It is a toxic protein that agglutinates red blood cells (Byrd-Leotis et al., 2015). The effect of this self-produced toxic protein must be considered while using such phases for biological therapy (Watts, 2017). Pseudomonas phages GP100 and TC6 also contain hemagglutinin proteins (Tang et al., 2018). This study isolated and identified two new virulent phage strains, which increases our understanding of the biodiversity of phages that can be used as antibacterial agents.

## Conclusion

Two different strains of *P. aeruginosa* phages were isolated. Although these belong to the same family, have the same host, and many other similarities, they showed distinct differences in plaque spot, morphology, growth characteristics, and tail fibers. Both the new isolates can be used for phage therapy and are a promising tool for the control of *P. aeruginosa*. The findings of this study may expand the knowledge of phages infecting *P. aeruginosa*, and provide new phages that may control *P. aeruginosa* infection.

## Data availability statement

The data presented in the study are deposited in the NCBI repository, accession number MZ727202.1 (PPAY) and MZ727332.1 (PPAT).

## Author contributions

NY wrote the first draft of the manuscript and carried out experiments. YX defined the research theme. WS, YY, ZH, LC, XB, ZX, and ZC designed the methods and experiments, analyzed the data, and interpreted the results. SZ, WJ, and LY provided experimental materials and equipments. YP and QZ wrote the manuscript. All authors contributed to the article and approved the submitted version.

## Funding

This work received support from National Natural Science Foundation of China (grant no. 42177414), Special Fund of China (grant no. AWS18J004).

## Conflict of interest

The authors declare that the research was conducted in the absence of any commercial or financial relationships that could be construed as a potential conflict of interest.

## Publisher’s note

All claims expressed in this article are solely those of the authors and do not necessarily represent those of their affiliated organizations, or those of the publisher, the editors and the reviewers. Any product that may be evaluated in this article, or claim that may be made by its manufacturer, is not guaranteed or endorsed by the publisher.

## Supplementary materials

The Supplementary materials for this article can be found online at: https://www.frontiersin.org/articles/10.3389/fmicb.2022.946251/full#supplementary-material
