# Supplementary material for: Isolation and characterization of two homolog phages infecting Pseudomonas aeruginosa
Source: Front Microbiol. 2022 Jul 14;13:946251. doi: 10.3389/fmicb.2022.946251 (PMC9348578; doi:10.3389/fmicb.2022.946251)
Supplement: Supplementary file 1 [file Data_Sheet_1.docx]

Isolation and characterization of two homolog phages infecting *Pseudomonas aeruginosa*

NIU Yuanyuan^1,2#^, YANG Xiaobo^2#^, WANG Shang^2^, YANG Yutong^2^, ZHOU Hongrui^2^, LI Chenyu^2^, XUE Bin^2^, ZHANG Xi^2^, ZHAO Chen^2^, SHEN Zhiqiang^2^, WANG Jingfeng^2^, LING Yun^1*^, YU Pingfeng^3^, QIU Zhigang^2,1*^

^1^College of Marine Ecology and Environment, Shanghai Ocean University, Shanghai 201306, China

^2^TianJin Institute of Environmental and Operational Medicine (Key Laboratory of Risk Assessment and Control for Environment & Food Safety), Tianjin 300050, China

^3^College of Environment and Resource Sciences, Zhejiang University, Zhejiang Hangzhou 310058, China

*** Correspondence:**QIU Zhigang
zhigangqiu99@gmail.com

LING Yun

[447417831@qq.com](mailto:447417831@qq.com)

Keywords: Bacteriophages_1_, *Pseudomonas aeruginosa*_2_, Genome_3_, Tail fiber protein_4_, Phage therapy_5_

Supplementary Information

Table S1. Host ranges of phage PPAT and PPAY on Pseudomonas aeruginosa strains

| **Strain** | **Spot testing** |
| --- | --- |
| Pseudomonas aeruginosa PAO1 (ATCC 15692) | + |
| Pseudomonas aeruginosa (ATCC 9027) | - |
| Pseudomonas aeruginosa (cmccb 10101) | - |
| Pseudomonas aeruginosa (cmccb 10102) | - |
| Pseudomonas aeruginosa (cmccb 10211) | - |
| Pseudomonas aeruginosa (ATCC 15442) | - |
| Pseudomonas aeruginosa (ATCC 27853) | - |


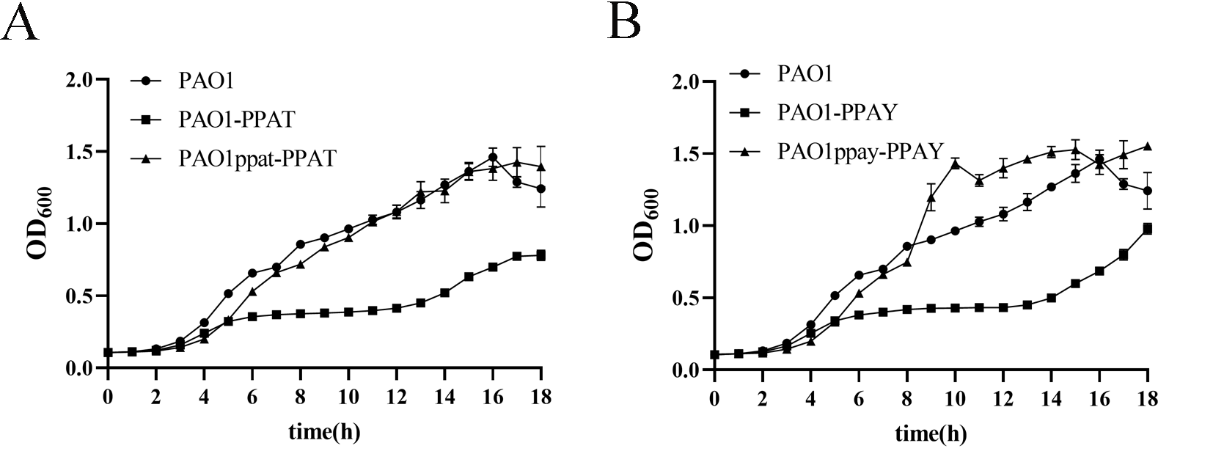
 **Figure S1. Growth curves of PAO1 and its phage-resistant strains under different conditions.** PAO1 is a wild type strain of Pseudomonas aeruginosa. PAO1ppat was a PPAT-phage-resistant mutant and PAO1ppay was a PPAY-phage-resistant mutant. PAO1 and its phage-resistant strains were infected with phage (MOI=100), respectively. Sample OD600 was measured every 1 h for 18 h using a fully automated growth curve analyzer (BIOSCREEN C0 PRO).
